# Metabolic regulation of ethanol-type fermentation of anaerobic acidogenesis at different pH based on transcriptome analysis of *Ethanoligenens harbinense*

**DOI:** 10.1186/s13068-020-01740-w

**Published:** 2020-06-03

**Authors:** Zhen Li, Yu Lou, Jie Ding, Bing-Feng Liu, Guo-Jun Xie, Nan-Qi Ren, Defeng Xing

**Affiliations:** State Key Laboratory of Urban Water Resource and Environment, School of Environment, Harbin Institute of Technology, 73 Huanghe Road, Nangang District, P.O. Box 2614, Harbin, Heilongjiang 150090 China

**Keywords:** Acidogenesis, Ethanol-type fermentation, Hydrogen-producing bacterium, *Ethanoligenens*, pH response, Transcriptome

## Abstract

**Background:**

Ethanol-type fermentation, one of the fermentation types in mixed cultures of acidogenesis with obvious advantages such as low pH tolerance and high efficiency of H_2_ production, has attracted widespread attentions. pH level greatly influences the establishment of the fermentation of carbohydrate acidogenesis by shaping community assembly and the metabolic activity of keystone populations. To explore the adaptation mechanisms of ethanol-type fermentation to low pH, we report the effects of initial pH on the physiological metabolism and transcriptomes of *Ethanoligenens harbinense*—a representative species of ethanol-type fermentation.

**Results:**

Different initial pH levels significantly changed the cell growth and fermentation products of *E. harbinense*. Using transcriptomic analysis, we identified and functionally categorized 1753 differentially expressed genes (DEGs). By mining information on metabolic pathways, we probed the transcriptional regulation of ethanol–H_2_ metabolism relating to pH responses. Multiple pathways of *E. harbinense* were co-regulated by changing gene expression patterns. Low initial pH down-regulated the expression of cell growth- and acidogenesis-related genes but did not affect the expression of H_2_ evolution-related hydrogenase and ferredoxin genes. High pH down-regulated the expression of H_2_ evolution- and acidogenesis-related genes. Multiple resistance mechanisms, including chemotaxis, the phosphotransferase system (PTS), and the antioxidant system, were regulated at the transcriptional level under pH stress.

**Conclusions:**

*Ethanoligenens* adapted to low pH by regulating the gene expression networks of cell growth, basic metabolism, chemotaxis and resistance but not H_2_ evolution-related genes. Regulation based on pH shifts can represent an important approach to establish and enhance ethanol-type fermentation. The complete gene expression network of ethanol fermentative bacteria for pH response provides valuable insights into the acidogenic fermentation, and offers an effective regulation strategy for the sustainable energy recovery from wastewater and solid waste.
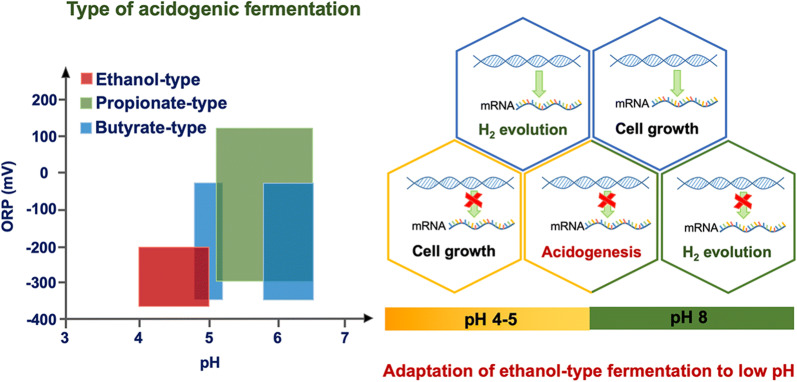

## Background

Anaerobic biotechnology integrates waste treatment and the generation of sustainable bioenergy and byproducts [1–3]. Anaerobic digestion (AD) as a form of anaerobic biotechnology includes four major phases: organic hydrolysis, acidogenesis, homoacetogenesis, and methanogenesis. The three well-known fermentation types in mixed cultures of acidogenesis are classified as butyric acid-type, propionic acid-type, and ethanol-type based on their liquid end products [4–6]. Products of ethanol-type fermentation mainly include ethanol, acetate, H_2_, and CO_2_, which are beneficial to subsequent methanogenesis. Ethanol can be converted to methane and acetate by syntrophic association of ethanol-oxidizing syntrophs and methanogens based on interspecies H_2_ transfer [3–5]. Ethanol-type fermentation also tolerates low pH values (e.g., the reactor operating with pH maintained at 4.0–4.5), representing a significant advantage to enhance acidogenesis and avoid the accumulation of propionic fermentation under higher H_2_ pressure [4–6]. Thus, it is important to understand metabolic regulation and community assembly in ethanol-type fermentation to improve the efficiency of AD.

Early studies indicated that ethanol-type fermentation prevailed in anaerobic reactors with pH of 4.0–5.0 and an oxidation reduction potential (ORP) of < − 200 mV [5, 6]. A representative genus of ethanol-type fermentation is *Ethanoligenens*, which was isolated from a hydrogen-producing continuous stirred-tank reactor (CSTR) [7]. *Ethanoligenens* generates ethanol, acetate, H_2_, and CO_2_, and these intermediates can be used for the production of high-value biochemicals and biopolymers [7–10]. Compared with the representative genus *Clostridium* of butyric acid-type fermentation, *Ethanoligenens* has characteristics of both ethanol–H_2_ co-production and acidophilia, which endows potential for further synergy with methanogens, as well as special autoaggregation and coaggregation abilities that enable the formation of anaerobic granule or biofilm in anaerobic fermentation processes [3, 5, 11, 12]. *Ethanoligenens* also serves as an important microbial intermediate in AD processes given that its metabolites (ethanol, acetate, H_2_, and CO_2_) can be further utilized by hydrogenotrophic and acetoclastic methanogens, acetogens, denitrifiers, iron and sulfate reducers, and syntrophs as substrates, electron donors, or energy carriers [13–17]. Given its importance in environmental biotechnology and ecological significance, *Ethanoligenens* exhibits considerable potential as a fermenter.

pH has a substantial influence on anaerobic processes and is not only essential for enrichment of dominant populations in different types of acidogenesis but also mediates the growth and metabolism of microorganisms that have different physiological and ecological characteristics [18–21]. Several studies have indicated that pH is important for stability and efficiency of AD systems and significantly influences the interactions between anaerobic fermentation bacteria and partner microorganisms, further changing fermentation patterns [11, 22]. Ren et al. reported for the first time that different fermentation types in mixed cultures can be established through pH control, and revealed the two-dimensional niches of pH and ORP for fermentation types of acidogenesis [4]. A relatively high pH (> 5.5) promotes acidogenesis via butyric acid-type and mixed acid-type fermentation, whereas a lower pH (4.0–4.5) enhances hydrogen generation by ethanol-type fermentation [4–6]. Changes in pH also directly contribute to the conversion from propionic acid-type fermentation to butyric acid-type or ethanol-type fermentation [6, 18, 23, 24]. Microbial community analyses based on the 16S rRNA gene and [FeFe]-hydrogenase gene (*hyd*) showed that microbiomes of hydrogen-producing reactors could be shaped by adjusting pH, and different dominant populations resulted in establishment of different fermentation types [5, 6].

From the cellular perspective, pH influences the activity of intracellular enzymes and further impacts metabolic pathways of bacteria; pH also affects the structure of cell membranes, changes the affinity of enzymes for their substrate, and affects the ability of bacteria to absorb substrates [11, 25, 26]. A transcriptomic study on *Clostridium thermocellum* (a fermentative anaerobic thermophile) showed that the ATP synthase gene was up-regulated and ATP-utilizing enzyme-encoding genes were down-regulated at pH 6.24, compared to pH 6.98 [27]. However, few studies have reported effects of pH on the metabolic regulation mechanisms of anaerobic fermentation bacteria at the gene expression level, where microorganisms essentially regulate gene expression to coordinate metabolic reactions in response to the environment [28]. Therefore, identifying key functional genes that affect metabolic networks is critical to design regulatory strategies for enhancing targeted gene expression at different pH.

In this study, we compared differences in metabolites of ethanol fermentative *E. harbinense* under different initial pH conditions. In doing so, we provide a global transcriptomic perspective of molecular mechanisms involved in metabolic regulation and pH stress responses of representative taxa involved with ethanol-type fermentation. We propose a pH control strategy to enhance ethanol–H_2_ co-producing acidogenesis.

## Results

### Effect of initial pH on the fermentation of *E. harbinense*

**Fig. 1:**
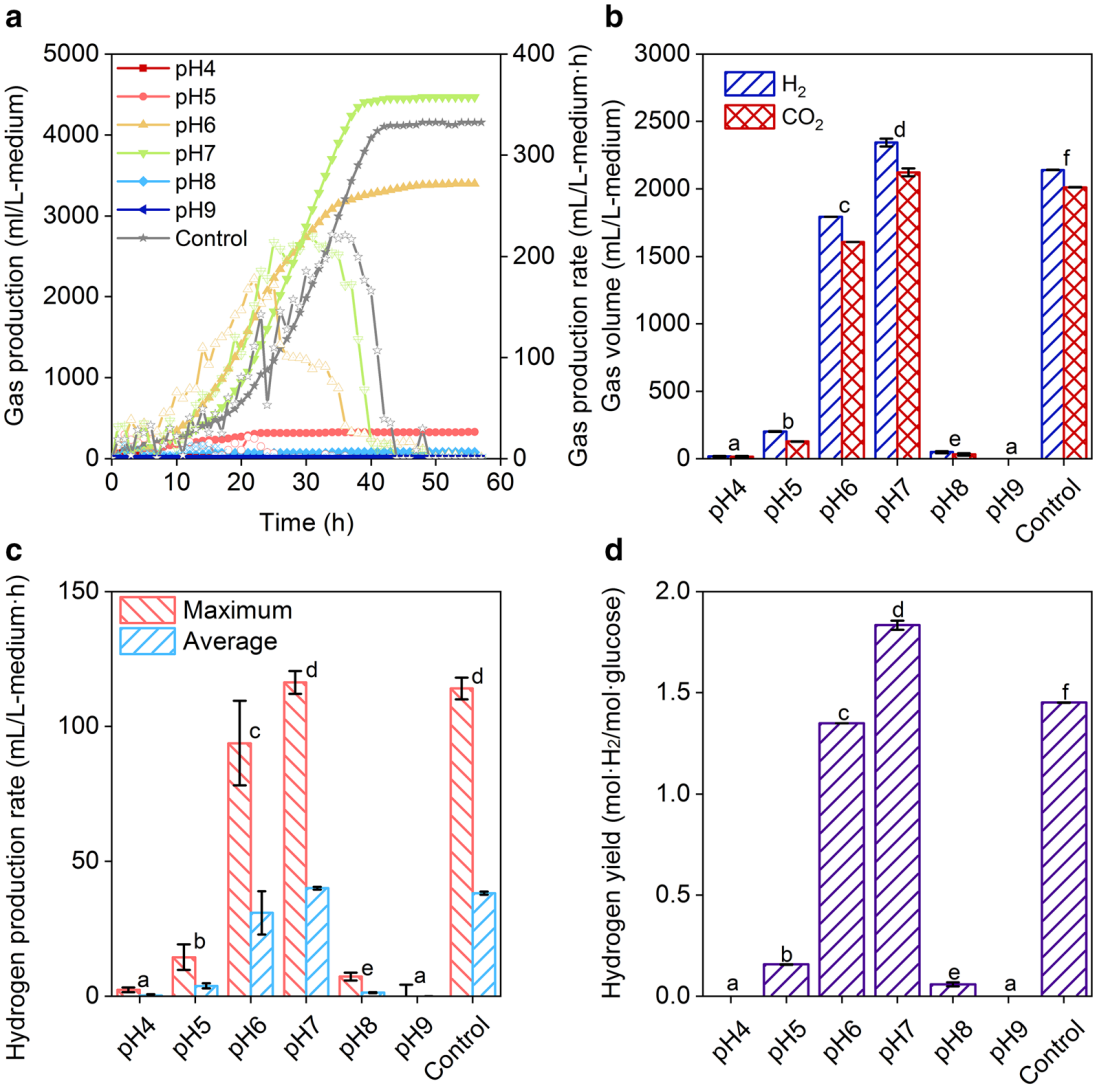
Gas production by *E. harbinense* at different initial pH values. **a** Gas production, data of gas production correspond to the lines marked by solid symbol while data of gas production rate correspond to the lines marked by hollow symbol; **b** gas composition; **c** maximum and average hydrogen production rate; and **d** hydrogen yield. Significant differences between data at different pH are shown (*p* < 0.05 or *p* < 0.01, one-way ANOVA, *n* = 3–8)

The fermentation metabolites of *E. harbinense* were mainly composed of ethanol, acetic acid, H_2_, and CO_2_ (Fig. 1). Significant differences in gas production and the gas production rate of *E. harbinense* were noted at different initial pH values but not between pH 7 and control (one-way ANOVA, *p* < 0.05, *n* = 3–8). Cumulative gas production at pH 7 (4465.80 ± 11.32 mL/L-medium) or control (4153.2 ± 9.37 mL/L-medium) was slightly higher than that at pH 6 (3397.51 ± 87.24 mL/L-medium). A higher or lower initial pH inhibited gas production (Fig. 1). *E. harbinense* produced 79.45 ± 1.27 mL/L-medium gas at pH 8 and no detectable gas at pH 9. Lower pH also thwarted gas production of *E. harbinense*, and the cumulative gas volumes at pH 4 and pH 5 were only 32.06 ± 4.42 and 328.13 ± 9.37 mL/L-medium, respectively, indicating that the suitable initial pH conditions for *E. harbinense* H_2_-metabolism were from 6 to 7 (Fig. 1a). In addition, the highest gas production rate was obtained at pH 7 (221.71 ± 8.13 mL/L-medium·﻿h), which was similar to that of control (221.45 ± 6.77 mL/L-medium·﻿h) and higher than that at pH 6 (177.83 ± 29.79 mL/L-medium·﻿h) and other pH conditions (Fig. 1a).

Significant differences in cumulative hydrogen production, hydrogen production rate, and hydrogen yield of *E. harbinense* were noted at different initial pH values (one-way ANOVA, *p* < 0.05, *n* = 3-8). The highest cumulative hydrogen production of 92.75 ± 3.11 mM (2343.75 ± 28.67 mL/L-medium) was at pH 7 compared to 70.94 ± 0.02 mM at pH 6 and 84.71 ± 0.06 mM at control (Fig. 1b). The maximum hydrogen production rates at pH 7 and pH 6 were 4.60 ± 0.17 mM/L-medium·h (116.35 ± 4.27 mL/L-medium h) and 3.71 ± 0.62 mM/L-medium﻿·h, respectively (Fig. 1c). The hydrogen proportions of different initial pH groups ranged from 51.55 to 61.24%. The hydrogen yields at pH 6, 7, and control were 1.35 ± 0.01, 1.83 ± 0.02, and 1.45 ± 0.01 mol-H_2_/mol-glucose, respectively, which were much higher than that at other initial pH, suggesting that the optimum pH for hydrogen production by *E. harbinense* was approximately 7 (Fig. 1d).

**Fig. 2:**
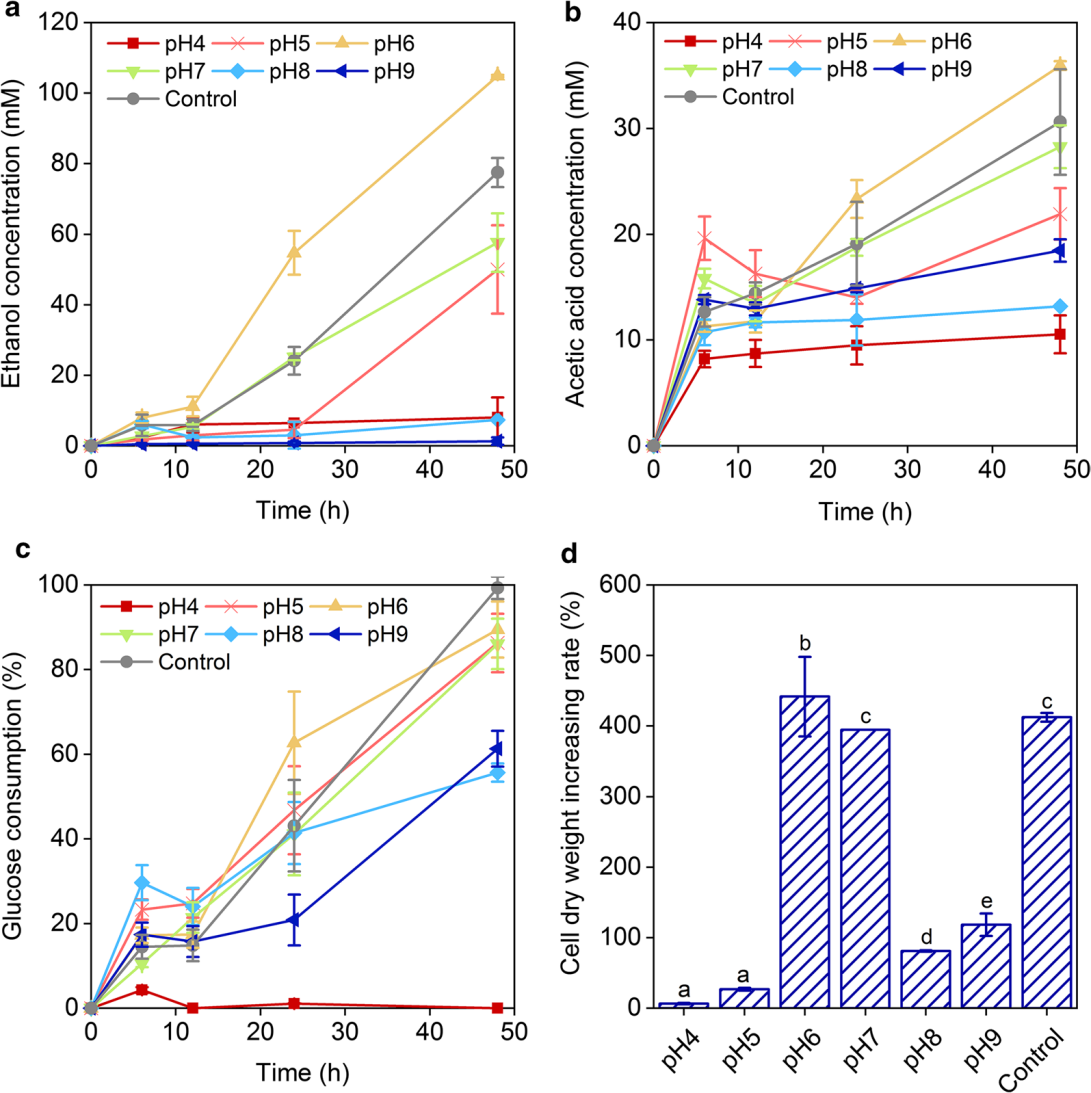
Initial pH effect on glucose consumption, cell growth, and fermentation products of *E. harbinense*. **a** Ethanol concentration; **b** acetic acid concentration; **c** glucose concentration; and **d** cell dry weight increase. Significant differences between data at different pH are shown (*p* < 0.05 or *p* < 0.01, one-way ANOVA, *n* = 3–8)

The final ethanol concentration at pH 6 was 104.90 ± 0.27 mM, which was higher than that at pH 7 (57.65 ± 8.30 mM) and pH 5 (50.02 ± 12.55 mM), and only small amounts of ethanol were detected at pH 4 (8.06 ± 5.65 mM), pH 8 (7.32 ± 0.03 mM), and pH 9 (1.34 ± 1.08 mM) (Fig. 2a). No significant differences in the final ethanol concentrations at pH 7 and control were noted (one-way ANOVA, *p* > 0.05, *n* = 3–8). The final acetate concentrations at pH 5, pH 6, and pH 7 were 21.92 ± 2.45, 35.94 ± 0.43, and 28.27 ± 2.01 mM, respectively, which were higher than those at pH 4 (10.54 ± 1.79 mM), pH 8 (13.18 ± 0.01 mM), and pH 9 (18.45 ± 1.06 mM). No significant differences were observed in the final acetate concentrations of *E. harbinense* at pH 5, pH 6, pH 7, and control (one-way ANOVA, *p* > 0.05, *n* = 3–8) (Fig. 2b). The accumulation of acetic acid caused a continual decrease in the pH value of fermentation broth, which eventually stabilized at 3.81–4.16, except at pH 8 (7.11) and pH 9 (9.22) (Additional file 1: Figure S1). The utilization of glucose was significantly different at different pH values (Fig. 2c). The values were 86.07 ± 9.55%–89.48 ± 0.78% at the end of fermentation at pH 5–7, which were much higher than the values of 55.65 ± 16.07% (pH 8) and 61.33 ± 15.29% (pH 9). At pH 4, glucose was essentially not being used (Fig. 2c). Cell dry weights at the end of fermentation reflected growth of *E. harbinense* at different initial pH conditions (Fig. 2d). Cells at initial pH 6 increased in mass by greater than fourfold (441.70 ± 56.63%), a value greater than those obtained at pH 7 (394.65 ± 0.09%) and pH 6.68 (412.35 ± 6.34%). *E. harbinense* grew slowly under suboptimal pH conditions (6.37 ± 1.00%, 26.71 ± 2.09%, 80.91 ± 1.01%, and 118.25 ± 15.86% at pH 4, pH 5, pH 8, and pH 9, respectively).

These results indicated that the initial pH significantly influenced both growth and metabolism of *E. harbinense,* and a pH value of 6–7 optimally facilitated metabolic and reproductive capacity. Extreme initial pH values inhibited growth and metabolism of *E. harbinense*.

### Transcriptome landscape of *E. harbinense* under different initial pH conditions

**Fig. 3:**
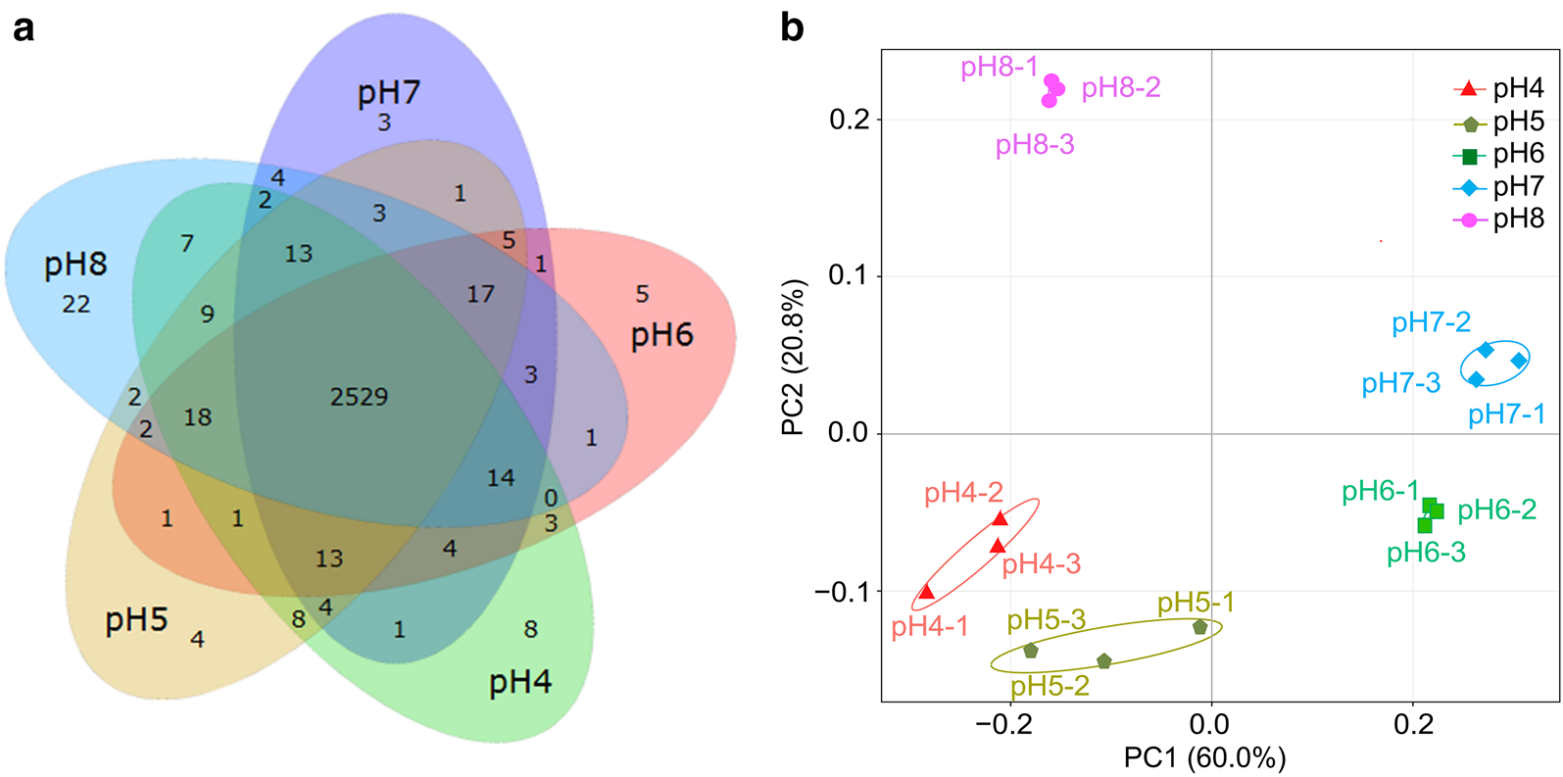
Gene expression analysis of *E. harbinense* under different initial pH conditions. **a** Venn diagram of gene expression; **b** principal component analysis

Over 2630 genes were identified in *E. harbinense* transcriptomes under different initial pH conditions, and the mean alignment rate with the *E. harbinense* genome was 93.07%. The Pearson correlation coefficient revealed expression similarities in three duplicates of each sample (*R*^2^ > 0.95) (Additional file 1: Figure S2), indicating that RNA-seq data were reproducible and reliable. A Venn diagram showed overlapping numbers of expressed genes under different initial pH conditions, and 2529 genes were expressed in all five groups. According to PCA, the similarity of transcriptomes between pH 6 and pH 7 as well as pH 4 and pH 5 was high; transcriptomes at pH 8 exhibited considerable differences compared with those at other pH levels (Fig. 3).

**Fig. 4:**
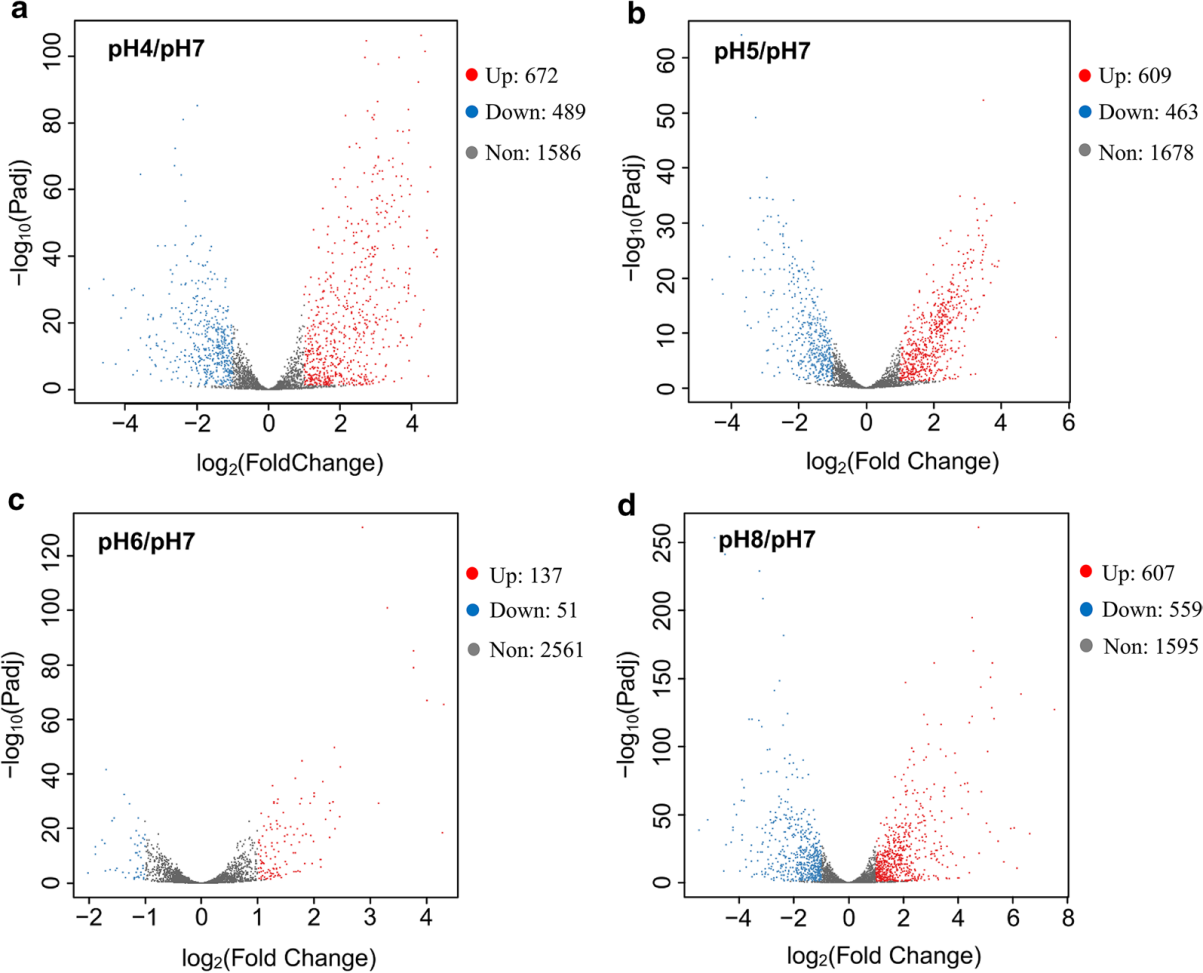
Differential gene expression (DEGs) volcano plot of *E. harbinense* under different initial pH conditions. The colors of DEGs represent the following: significantly up-regulated (red), significantly down-regulated (blue), and unregulated (gray)

A total of 1753 DEGs were found in the transcriptomes of five groups with different initial pH conditions. Compared with the transcriptome at pH 7, 672 genes were up-regulated and 489 genes were down-regulated in the transcriptome at pH 4. The highly up-regulated genes (log_2_(FoldChange) ≥ 4) at pH 4 included four phosphotransferase system (PTS) sugar transporter genes (Ethha_0815, Ethha_0816, Ethha_0817, and Ethha_0818), which are important for the carbohydrate uptake of bacteria (Additional file 1: Figure S4). Most down-regulated genes (log_2_(FoldChange) ≤ − 4) at pH 4 were hypothetical proteins having no functional annotation. The number of DEGs at pH 5 and pH 8 was slightly smaller than that at pH 4. However, the transcriptome at pH 8 had more highly regulated genes (log_2_(FoldChange) ≥ 4 or ≤ − 4). The highest up-regulated gene at pH 8 (183 times compared with that at pH 7, log_2_(FoldChange) = 7.52) was a thioredoxin gene (Ethha_0126) that is important in oxidative stress responses for acid resistance of bacteria [11] and is closely related with alkali resistance of *E. harbinense*. A flagellin gene (Ethha_2548) was highly down-regulated (log_2_(FoldChange) = − 4.56) at pH 8. Transcriptomes at pH 6 and pH 7 exhibited similar gene expression patterns; only 137 genes were up-regulated and 51 genes were down-regulated at pH 6, in contrast to those at pH 7 (Fig. 4). Genes with increased regulated expression at pH 6 included a series of transporter genes, mainly PTS system sugar transporter genes, which were also highly up-regulated at pH 4.

### Functional annotation of DEGs in *E. harbinense*

**Fig. 5:**
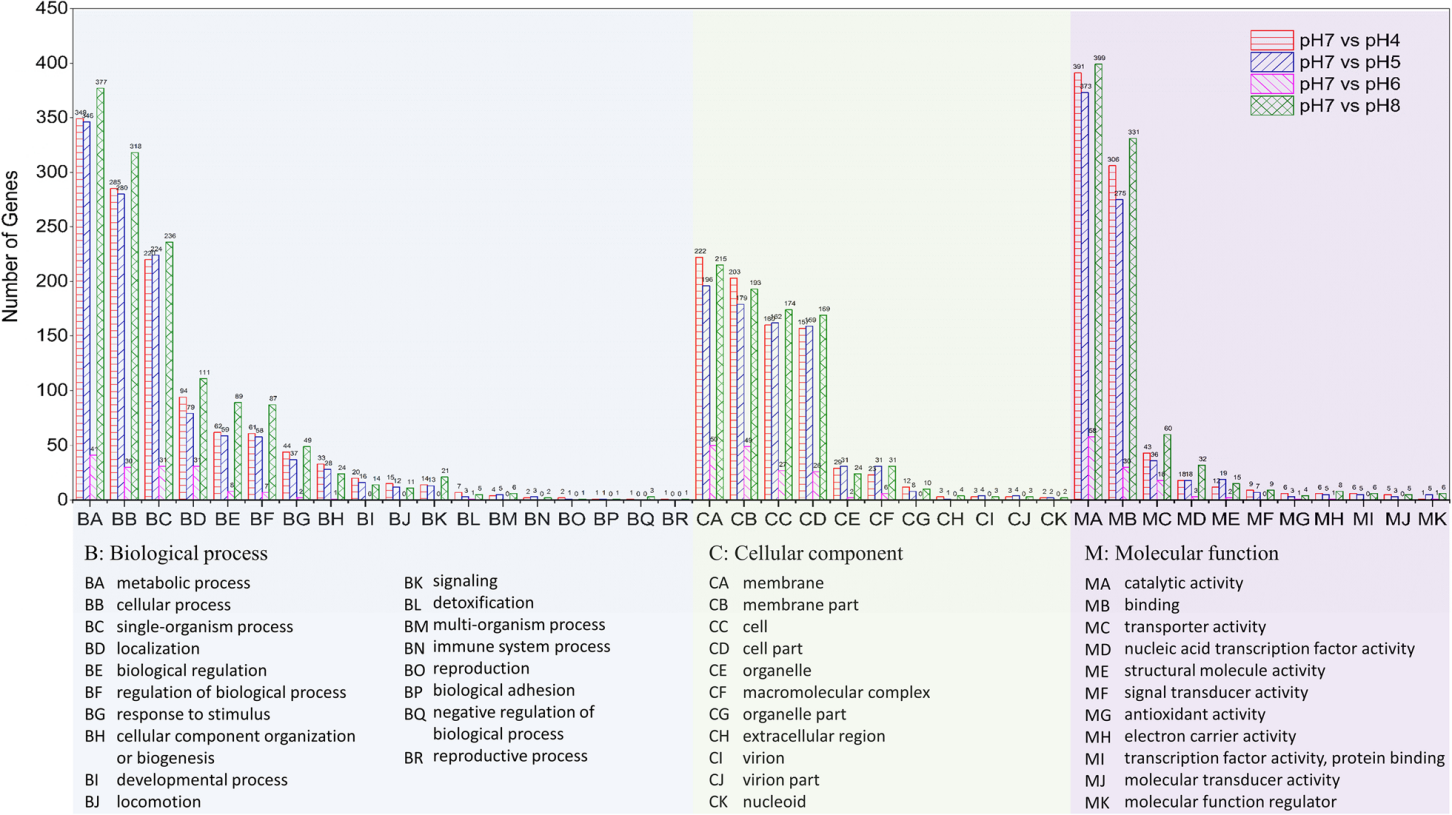
DGE clusters of GO enrichment of *E. harbinense* under different initial pH conditions. Different background colors correspond with GO terms: biological process (blue), cellular component (green), and molecular function (purple)

According to the clusters in the Gene Ontology (GO) enrichment analysis, DEGs were further clustered into 40 groups with different functions (Fig. 5). These DEGs were distributed mainly in the following categories: metabolic process, cellular process, and single-organism process for biological process; membrane and cell for cellular component; and catalytic activity and binding for molecular function. These changes in fundamental gene expression affect the most essential activities of bacteria.

**Fig. 6:**
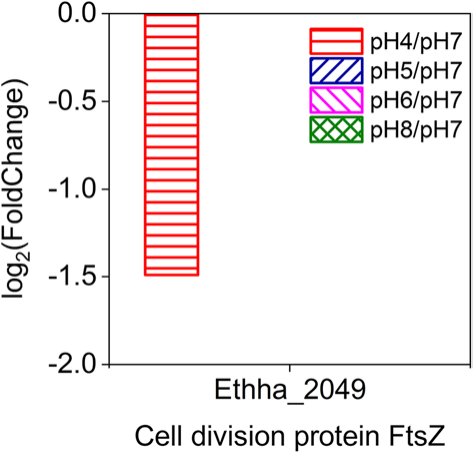
Expression of the cell division protein FtsZ in *E. harbinense* involved in reproductive processes

Expression of some genes involved in specialized pathways, such as stimulus response, locomotion, signaling, electron transfer, detoxification, antioxidation, reproduction, and biological adhesion, was also altered based on pH (Additional file 1: Table S1). Expression of Ethha_2049, a gene encoding the cell division protein FtsZ that participates in the reproduction process, was significantly down-regulated at pH 4 (log_2_ (Fold Change) = − 1.49) (Fig. 6). Genes encoding chemotaxis system-related proteins altered their expressions in response to different extracellular pH conditions, including two HAMP domain-containing histidine kinase (chemoreceptors) genes (Ethha_1123 and Ethha_1927), chemotaxis protein CheA/CheW/CheB genes (Ethha_2543, Ethha_2545 and Ethha_2546), and multiple flagella-related genes. Among flagella-related genes, *fliD* (Ethha_2541), a gene encoding flagellar filament capping protein FliD and participating in biological adhesion processes, was differentially expressed under varied initial pH conditions, potentially explaining the differences in both cell motility and autoaggregation of *E. harbinense*. A group of genes encoding proteins with antioxidant activity were differentially expressed under varied pH conditions, for example, the peroxiredoxin gene (Ethha_0300), thioredoxin-disulfide reductase gene (Ethha_0354), and glutathione peroxidase gene (Ethha_2772), which might be important to maintain redox balance for cell growth.

**Fig. 7:**
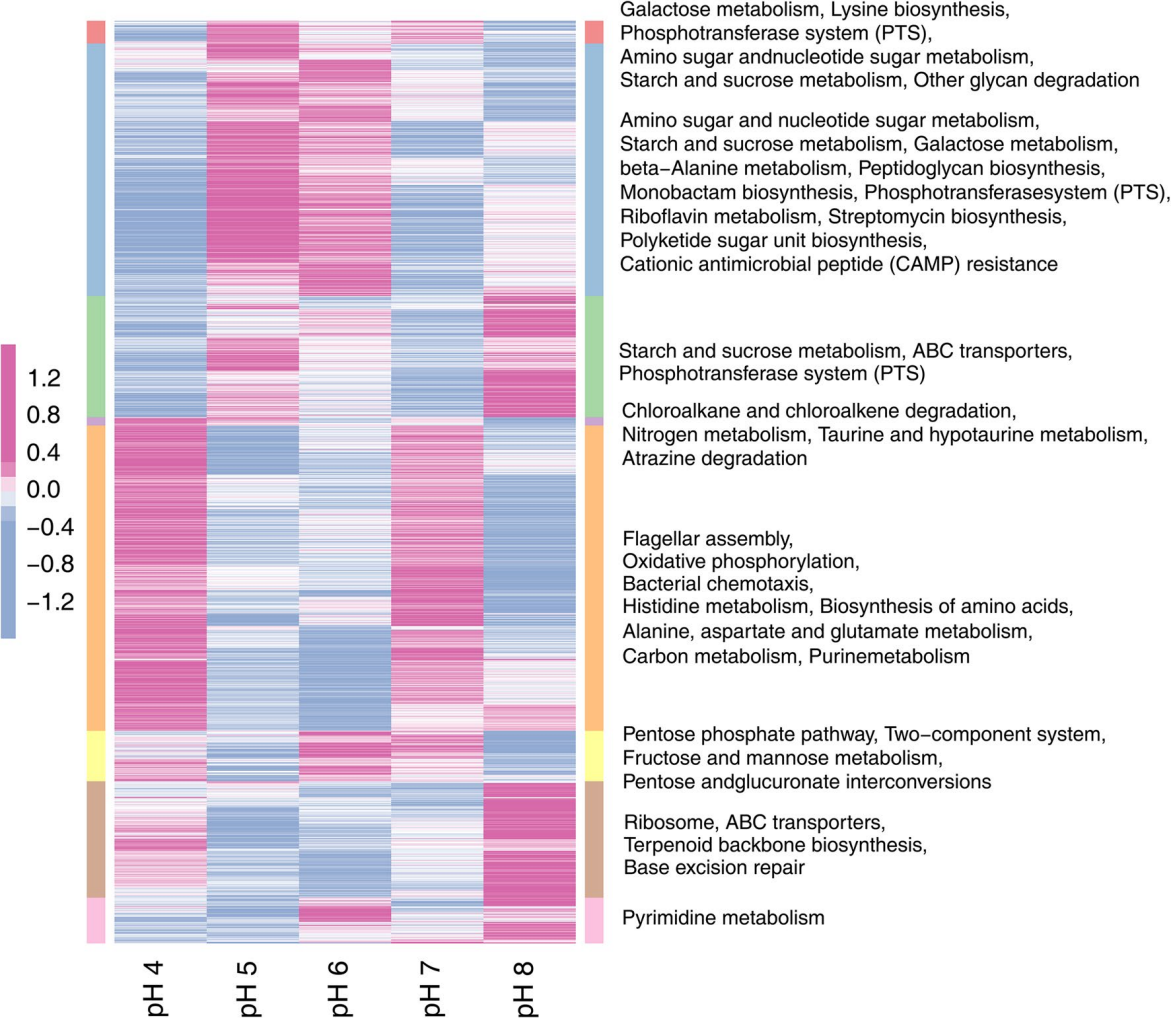
Heatmap and KEGG pathway enrichment of DEGs of *E. harbinense* under different initial pH conditions. Gene expression data were analyzed by hierarchical clustering and grouped into 8 clusters that were associated with significantly enriched KEGG terms (right) (FDR *q* < 0.01). The darker pink represents higher relative abundance and expression levels of genes, whereas darker blue represents lower relative abundance and expression levels of genes

Eight major clusters of DEGs were classified according to similarities in gene expression patterns and further analyzed by KEGG pathway enrichment (Fig. 7). Genes enriched in KEGG analysis participated in activities such as sugar metabolism, the PTS system, flagellar assembly, oxidative phosphorylation, and bacterial chemotaxis (consistent with results of GO enrichment). Other genes involved in biological processes that are extremely important for cell growth were also enriched, including protein-coding genes of carbon metabolism, nitrogen metabolism, amino acids metabolism/biosynthesis, purine/pyrimidine metabolism, ABC transporters, ribosome, two-component system, and base excision repair. The results indicated that the initial pH caused dramatic changes in the gene expression profiles of *E. harbinense*. Different extracellular pH conditions affected activity of glycolytic enzymes, induced energy and metabolism fluctuations in cells, and triggered a series of pH stress responses.

### Ethanol–H_2_ co-metabolism of *E. harbinense* under different initial pH conditions

**Fig. 8:**
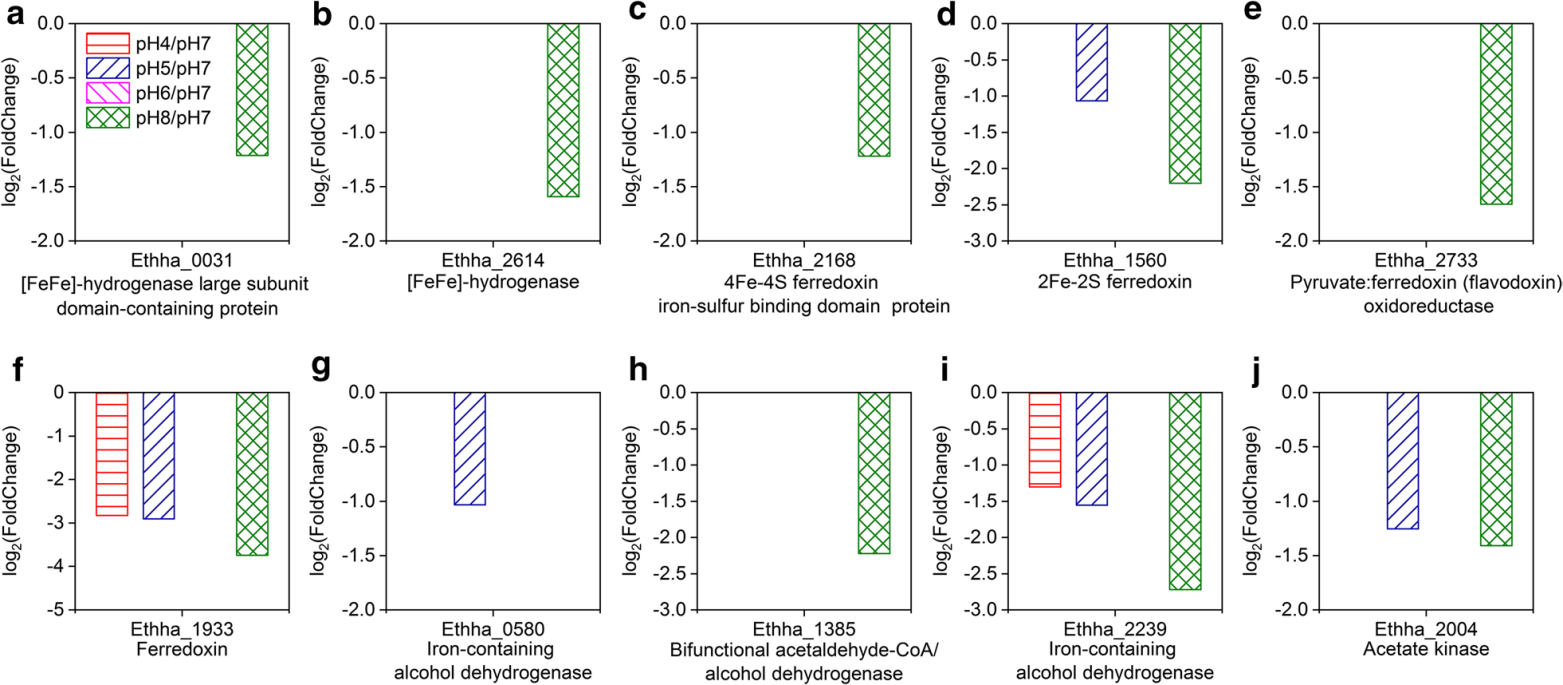
DEGs of *E. harbinense* involved in [FeFe]-hydrogenases (**a**, **b**), ferredoxins (**c**–**f**), alcohol-dehydrogenases (**g**–**i**), and acetate kinase (**j**)

Some of the key genes involved in the ethanol–H_2_ co-production pathway of *E. harbinense* exhibited gene expression alterations under different initial pH conditions. The enzymatic catalytic reaction processes for hydrogen production were influenced by high pH at the transcription level. Compared with the transcriptome at pH 7, two [FeFe]-hydrogenase genes (Ethha_0031 and Ethha_2614) were significantly down-regulated at pH 8 (log_2_(FoldChange) values were −1.21 and −1.59, respectively), but neither of them showed a significant difference in gene expression under acidic conditions (Fig. 8a, b). Genes encoding ferredoxins that function in electron transfer were also down-regulated at pH 8, including Ethha_1933, Ethha_1560, and Ethha_2168 (log_2_ (Fold Change) values were − 3.74, − 2.20, and − 1.22, respectively) (Fig. 8c–e). Ethha_2168 is a 4Fe–4S ferredoxin iron–sulfur binding domain protein-encoding gene, which is essential for formation of the core domain of the H-cluster in [FeFe]-hydrogenase (Hyd). The significantly down-regulated expression of Ethha_2168 at pH 8 might reduce electron transport and affect the H_2_ generation process. In addition, the expression of Ethha_2733, which encodes a pyruvate:ferredoxin (flavodoxin) oxidoreductase (PFOR), was down-regulated (log_2_(FoldChange) = − 1.66) at pH 8. These results indicate that fewer pyruvate molecules were decomposed to acetyl-coenzyme A (acetyl-CoA) by oxidative decarboxylation, potentially leading to decreased hydrogen production by *E. harbinense* (Fig. 8f).

Effects of the initial pH on *E. harbinense* also resulted in changes in ethanol and acetate acid generation. Three of 7 genes encoding alcohol dehydrogenase proteins were down-regulated at pH 4 or pH 8; thus, ethanol yields of *E. harbinense* were reduced under these conditions (Fig. 8g–i). The acetate kinase gene (Ethha_2004) was down-regulated at both pH 5 and pH 8 (the log_2_(FoldChange) values were − 1.25 and − 1.41, respectively), which decreased the formation of acetic acid (Fig. 8j).

### Gene expression changes of new transcripts of *E. harbinense* under different initial pH conditions

New transcripts (*n* = 541) were predicted for all samples of *E. harbinense* YUAN-3 by RNA-seq. Among them, 32 were transcripts of new protein-coding genes, and 509 were non-coding RNAs. Seven of these new transcripts showed significant changes under different initial pH conditions (Additional file 1: Figure S5 and Table S2). Novel_C19, novel_C20, and novel_C28 exhibited similar expression patterns and were up-regulated at different pH conditions. The novel_C28 was involved in functional regulation of various processes, such as cell motility, signal transduction, and carbohydrate metabolism under different pH stresses, according to KEGG pathway analysis. As a membrane transport protein-encoding gene, the up-regulated expression of the novel_C19 might facilitate the rapid pH response of *E. harbinense*. However, another membrane transport protein-encoding gene, the novel_C12, down-regulated its expression at lower pH conditions (log_2_(FoldChange) values were −1.96 at pH 4 and −2.22 at pH 5, respectively). The expressions of novel_C16 and novel_C31 were down-regulated at different pH conditions, which might influence metabolism of carbohydrates and nucleotides and biosynthesis of other secondary metabolites. All 7 differentially expressed new genes identified in *E. harbinense* contribute to elucidating the regulation network of the pH response of *E. harbinense*, and these findings need to be further confirmed by strategies such as gene knockout, gene silencing, or gene overexpression.

## Discussion

### Fast start-up of ethanol-type fermentation by pH regulation

In practical applications, a rapid and reliable start-up method is required to efficiently enrich suitable populations in reactors and establish AD systems with stable performance [29]. Heat shock treatment of mixed inoculum is often used to accelerate enrichment of spore-forming H_2_-producing bacteria and to inhibit methanogens. This treatment is ineffective for enrichment of non-spore-forming H_2_-producing bacteria (such as *Ethanoligenens*) [19]. Low pH is beneficial for establishing ethanol-type fermentation and suppressing butyrate or propionate fermentation types and methanogenesis [4–6]. *Ethanoligenens* is the representative hydrogen-producing genus of ethanol-type fermentation, which is often predominant population in continuous-flow hydrogen-producing reactors operated at low pH values (4.0–4.5) [5]. Assessment of the influence of pH on the metabolic regulation of *Ethanoligenens* can help us better understand its functions in acidogenesis and provide more information to target an optimal pH value of reactor operations to yield the best possible performance of ethanol-type fermentation. In actual operations of anaerobic reactors, start-up conditions with low initial pH values lead to a long enrichment time of ethanol fermentation bacteria [30]. Here, we proposed a strategy of screening ethanol fermentation bacteria by switching pH to achieve both bacterial bio-enhancement and fast start-up of anaerobic reactors. Microorganisms working in this hypothetical hydrogen-producing reactor may aggregate to form granular sludge. This effect may rely on the autoaggregation ability of *Ethanoligenens* or how these bacteria form biofilms by biological immobilization of fillers. These mechanisms still need to be further validated.

### Influences of pH on growth and ethanol–H_2_ co-production

Essentially, bacteria respond to external pH stresses by regulating gene expression [31]. Understanding the regulation of metabolic networks and finding key functional genes that influence metabolic flux are helpful to coordinate anaerobe metabolites at acidic/alkaline pH. The appropriate initial pH of *Ethanoligenens* ranged from 6.0 to 7.0 with the most active expression of genes, whereas pH values that were too low or too high had different effects on the growth and metabolism of *Ethanoligenens* at the gene expression level. Results showed that low initial pH conditions inhibited the increase in cell dry weights (Fig. 2d), and the effects of low initial pH on bacterial growth of *E. harbinense* were mainly concentrated in reproductive processes. A cell division protein-encoding gene (*ftsZ*) (Ethha_2049) was down-regulated at pH 4 (Fig. 6), which might reduce cell proliferation since FtsZ plays a prominent role in cytokinesis of cell division [32, 33] and the concentrations of cellular FtsZ regulate the frequency of division [34]. Low initial pH had no significant effect on expression of the [FeFe]-hydrogenase gene (*hyd*), which plays a catalytic role in the final step of H_2_ production [3]. *E. harbinense* has 10 ferredoxins with electron transport activity, and 4 of them are 4Fe–4S ferredoxins, which are the core domain of the H-cluster of [FeFe]-hydrogenase [3]. The expression of only two of these ferredoxins (Ethha_1560 and Ethha_1933) was down-regulated at low pH, which might have slight effects on electron transfer and hydrogen production (Fig. 8d, f). The gene expression of Ethha_2733 was not significantly affected by low initial pH values (Fig. 8e). Ethha_2733 is a pyruvate:ferredoxin (flavodoxin) oxidoreductase (PFOR), a key enzyme that catalyzes oxidative decarboxylation of pyruvate to acetyl-coenzyme A (acetyl-CoA), which plays a decisive role in hydrogen production [3, 35]. Low pH-mediated inhibition of ethanol production was not as obvious as that on acetic acid production, which might due to down-regulated expression of only 2 out of all 7 alcohol dehydrogenase genes in *E. harbinense* [3]. This feature may have caused some compensatory effects on ethanol production by stable expression of other alcohol dehydrogenase genes. Conversely, gene expressions of most ethanol–H_2_ co-metabolic enzymes were down-regulated at high pH (Fig. 8) and resulted in no hydrogen production at pH 9 (Fig. 1c). Nevertheless, the cell dry weights slightly increased at higher pH conditions, and greater acetic acid yields were noted at pH 4 (Fig. 2b, d), suggesting that alkaline conditions had minimal influence on viability and acidogenesis of *E. harbinense*.

**Fig. 9:**
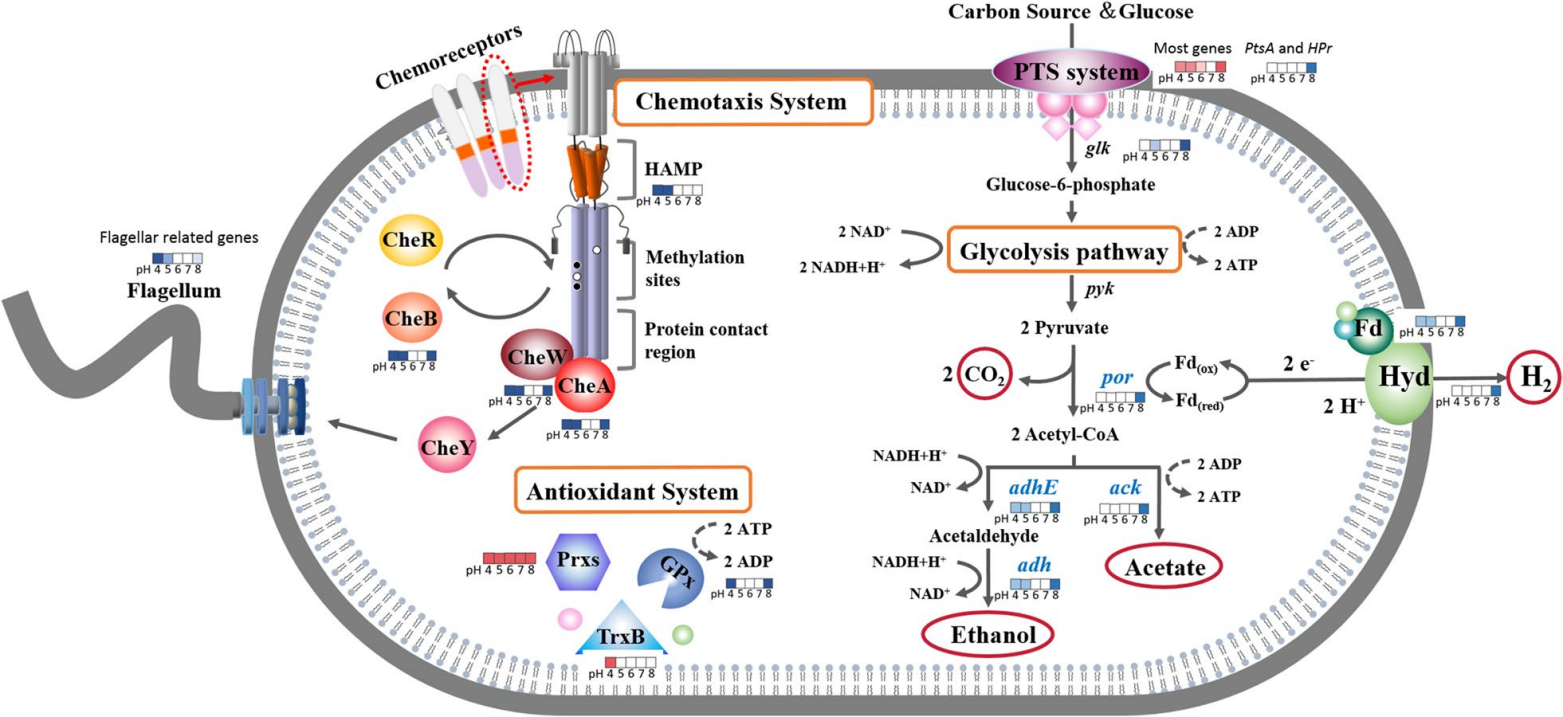
Proposed molecular response process and metabolic pathways of *E. harbinense* for pH-based responses. The colors in the rectangles indicated changes of gene expression under different initial pH conditions, the blue and red represent the down-regulation and the up-regulation, respectively

Carbohydrate is mainly oxidized through the EMP pathway and broken down into pyruvate, which is further oxidized into ethanol and acetic acid, and electrons are transferred to ferredoxins and [FeFe]-hydrogenases to generate H_2_ [3]. The production of liquid end products was generally followed by H_2_ production [12]. The glucose utilization by *E. harbinense* at pH 5–7 (86–89%) was similar, which was higher than those of pH 8 and pH 9 (56–61%) and of pH 4, which were consistent with the trend of gas production at different pH conditions. However, the molar ratios of ethanol to acetic acid of pH 5–7 were only ranged from 0.34 to 0.49, which were much lower than those of pH 4 (1.31), pH 8 (1.80), and pH 9 (13.79). Meanwhile, the transcriptomic results indicate that lots of genes related to cell growth and basic metabolisms were significantly down-regulated under unfavorable pH conditions. Low initial pH mainly affected cell growth and proliferation, but H_2_-metabolic enzyme activity was not altered. High pH mainly inhibited hydrogen production since the expression of H_2_ evolution-related genes was only down-regulated at pH 8, but had smaller effects on acetic acid generation and cell proliferation. Therefore, these findings suggest that hydrogen generation by *E. harbinense* not only depends on the activity of H_2_ evolution-related enzymes (such as hydrogenases and ferredoxins), but also on the availability of electrons transferred to [FeFe]-hydrogenase and the distribution of electron flow (Fig. 9).

As the dominant bacteria in ethanol fermentation, *E. harbinense* showed strong acid resistance in continuous-flow acidogenic reactors for H_2_ production at pH values less than 4.0 [7, 36]; however, it is difficult for *E. harbinense* to live in isolation and produce hydrogen in an overly acidic/alkaline environment in a pure culture system. *E. harbinense* tends to operate better as the dominant species in a microbial community compared with a pure culture, especially in extreme pH conditions, possibly due to syntrophic mechanisms with other bacteria. Further studies could focus on co-culture of *E. harbinen*se with other microorganisms and explore possible synergistic interaction mechanisms.

### pH effects on specific systems of *E. harbinense*

The pH level influences many biological processes of fermentative bacteria, such as enzyme reactions and interactions between proteins, that are critical for growth metabolism of bacteria [37]. Low pH can result in low cellular energy levels, which limit the ability of cells to maintain internal pH. The activity of enzymes in metabolic reactions will be restrained under high pH conditions, gradually affecting nutrient absorption by bacteria [37, 38]. Therefore, bacterial survival under unfavorable pH conditions induces protective responses, maintaining homeostasis of internal pH and preparing the cell to survive under extreme pH conditions in future exposure. Acidic or alkaline conditions inhibited growth and metabolism of *E. harbinense*, which activated a series of resistance mechanisms, including mechanisms of chemotaxis, the PTS system, and the antioxidant system, and affected hydrogen production by *E. harbinense* (Fig. 9).

The gene expression of most PTS sugar transporters was up-regulated potentially to increase carbon source uptake as responses to low or high pH conditions (Additional file 1: Figure S4). However, the expression of genes encoding PTS glucose transporter subunit IIA and IIB (Ethha_0722 and Ethha_0723, respectively) significantly down-regulated at pH 8, exhibiting negative effects on the growth and metabolism of *E. harbinense* (which was cultured using glucose as the carbon source). Phosphoenolpyruvate–protein phosphotransferase (PtsA) and HPr proteins are critical to govern hierarchal uptake of carbon sources, regulate glucose metabolism and catabolite repression pathways, and maintain cell activity of bacteria [39]. However, the expression of two PtsA-encoding genes (Ethha_0720 and Ethha_1484) and two HPr family phosphocarrier protein-encoding genes (Ethha_2064 and Ethha_0719) was significantly down-regulated at pH 8, which might cause glycometabolic disorders, aggravate metabolic inhibition, and influence cell growth and metabolism of *E. harbinense*. In addition, PtsA located on the bacterial surface has an additional adhesin function to mediate bacterial attachment to other cells [40]. According to the prediction results of protein subcellular localization, most PtsA of *E. harbinense* was cytoplasmic, and a few molecules might be located at cell walls or extracellularly. Cell wall-localized PtsA might be related to the autoaggregation and coaggregation ability of *E. harbinense*, which needs to be further verified in vivo and in vitro through biochemical and immunostaining methods.

Antioxidant genes were differentially expressed under different initial pH conditions, indicating that pH stress induced the oxidative stress response in *E. harbinense* (Additional file 1: Table S1). Ethha_0300, a peroxiredoxins (Prxs)-encoding gene of *E. harbinense*, was up-regulated in all pH conditions to protect cells against reactive oxygen species and potentially promote regulation of signal transduction [41, 42]. A thioredoxin-disulfide reductase-encoding gene (Ethha_0354) of *E. harbinense* was up-regulated at pH 4, and this gene might play a positive role in maintenance of redox homeostasis in cells to tolerate low pH [11, 43]. However, the expression of a glutathione peroxidase-encoding gene (Ethha_2772) was significantly down-regulated at pH 4 and pH 8, which might lead to inadequate detoxification in *E. harbinense* cells and cause cellular malfunctions responsible for several pathologies and aging [42, 44].

Chemotaxis systems are typical transmembrane sensory and signaling mechanisms of prokaryotes, which are ubiquitously used to perceive environmental stimuli, such as sugars, pH, temperature, and osmotic pressure, and to adjust to various environmental niches by controlling microbial cell motility toward favorable conditions [31, 45, 46]. The low pH (negative stimuli) changed the expression of two chemoreceptor-encoding genes (Ethha_1927 up-regulated and Ethha_1123 down-regulated) (Additional file 1: Table S1), potentially causing chemoreceptor accommodation and binding between periplasmic sensory domains and specific types of ligands transducing signals to the HAMP homodimer domain of chemoreceptors [45, 47–49]. Signals are then transduced through the cytoplasmic regions and interact with the associated kinase CheA and the adaptor protein CheW [50]. The expression of *cheA* (Ethha_2546) and c*heW* (Ethha_2545) in *E. harbinense* was down-regulated at low pH, and the inhibition of these proteins might subsequently pass the phosphoryl group to the response regulator CheY, which binds to flagellar motors to control flagellar rotation [46]. The expression of flagellar genes associated with CheY was down-regulated, potentially causing flagellar rotation and changes in the motion direction of *E. harbinense* in response to external pH stimuli (Fig. 9). Conversely, the chemoreceptor gene expression was unchanged. The expressions of *cheA* and *cheW* was directly affected (down-regulated) at high pH conditions and caused subsequent down-regulation in the expression of a series of flagellar-related genes. In addition, an adaptation system consisting of the methyltransferase CheR and the methylesterase CheB modulates the activity of chemoreceptors that enable chemotaxis systems to terminate the behavioral response according to background stimulation [45, 46]. The expression of the *cheB* (Ethha_2543) was down-regulated under both acidic and alkaline conditions, indicating that the chemotaxis systems of *E. harbinense* operated continuously under pH stress conditions. The above results indicate that *E. harbinense* has similar signaling regulatory mechanisms as *Bacillus subtilis*, which represents one of two chemotaxis modes (*Escherichia coli* is the opposite one) in which negative chemotactic stimuli inhibit activity of CheA, promote clockwise flagellar rotation, and cause random changes in the direction of movement [46]. To our knowledge, this is the first report of a chemotaxis pathway of the pH response of fermentative hydrogen-producing bacteria. To verify the chemotaxis pathway and the effects on ethanol–H_2_ co-production of *E. harbinense*, further studies should be performed using mutations in the HAMP region of receptors or gene knockout/overexpression of *cheA*/*cheW*/*cheB*.

## Conclusions

To reveal underlying mechanisms of adaptation of ethanol-type fermentation to low pH, the transcriptome dynamics of ethanol–H_2_ co-producing *Ethanoligenens* at different pH values were investigated. A total of 1753 DEGs of *Ethanoligenens* were identified under different initial pH conditions. Low initial pH mainly affected cell growth and proliferation, but the activity of H_2_-metabolic enzymes was not affected. High pH mainly inhibited hydrogen production and acidogenesis. PTS system-related genes changed their expression to maintain carbohydrate and energy uptake balance. Antioxidant mechanisms were stimulated by unsuitable pH to activate acid/alkali resistance mechanisms, and the chemotaxis system was regulated to perceive signals of extracellular pH stimuli and further alter the direction of cell motility. In this study, a complete gene expression network was constructed to interpret pH response mechanisms of hydrogen-producing bacteria, and the characteristics of ethanol-type fermentation and fermentative hydrogen production at low pH were described.

## Materials and methods

### Bacterial growth conditions and pH treatment

*Ethanoligenens harbinense* YUAN-3, isolated from the anaerobic activated sludge in a continuous-flow hydrogen-producing reactor and preserved in our laboratory [7], was cultivated anaerobically using a modified peptone/yeast extract/glucose (PYG) medium under an N_2_ atmosphere (ultra-high purity of 99.999%) at 35 °C with a shaking speed of 170 rpm. The modified PYG medium (per liter) contained 12.0 g glucose, 4.0 g peptone, 1.0 g yeast extract, 4.0 g NaCl, 1.5 g K_2_HPO_4_, 0.1 g MgCl_2_·6H_2_O, 0.5 g l-cysteine, 5 mL Wolfe’s vitamin mixture, and 5 mL trace mineral mixture [7].

*E. harbinense* in the logarithmic stage were collected by centrifugation (12,000×*g*, 1 min) and immediately inoculated into 200-mL anaerobic fermentation bottles that contained 100 mL of the modified PYG medium with different initial pH values (4, 5, 6, 7, 8, and 9). The initial pH was adjusted using 1 mol/L NaOH and 1 mol/L HCl. The salinity of medium was unified using 1 mol/L NaCl. The PYG medium without pH adjustment served as a control, and its initial pH was 6.68. Each group trial was conducted in triplicate.

### Fermentation product analysis

Gas production was recorded using a multi-channel respirometer (AER-208, Challenge Environmental Systems Inc., USA), and the produced gas was collected using 0.1-L gas bag (DeLin, China). Before the experiments, respirometer gas passages were treated with filter-sterilized and ultra-high pure N_2_ gas (99.999%) to remove the residual gas from previous tests. The gas component was analyzed using a gas chromatography (Agilent 7890, USA) by means of thermal conductivity detection, with ultrapure N_2_ (99.99%) as the carrier gas. VFAs and alcohols were measured using a gas chromatograph (Agilent 6890, USA) equipped with a flame ionization detector and a fused-silica capillary column (DB-FFAP) with N_2_ as the carrier gas. The glucose concentration was determined using a Glucose Detection Kit (Shanghai Rongsheng Biotech Co., Ltd., China). The pH was measured using a standard meter (PHSe3C, Yangguang Co. Ltd., China). Biomass was measured at the end of fermentation, and *E. harbinense* cells were collected by centrifugation at 4 °C (12,000×*g*, 10 min,) and freeze-dried using a vacuum freeze dryer (SP Scientific, UK) to measure cell dry weight. The hydrogen production rate (${\text{Q}}_{\text{H}_2}$, mL/L-medium h) was calculated based on the stable cumulative hydrogen production rate normalized to liquid volume. The hydrogen yield and utilization ratio of glucose were calculated as previously described [51, 52]. The hydrogen yield (${\text{Y}}_{\text{H}_2}$) was the molar value of hydrogen production normalized to the molar value of substrate consumption (mol H_2_/mol-glucose).

### RNA extraction and RNA-seq analysis

Samples for transcriptomic analysis were harvested at 24 h after inoculation via centrifugation (4 °C, 12,000×*g*, 5 min). Samples were immediately frozen in liquid nitrogen and stored at − 80 °C for further RNA extraction. The FastPrep-24 instrument (MP Biomedical, USA) was used to break bacterial cells. Total RNA was isolated using the FastRNA^®^ Pro Blue Kit (MP Biomedicals, USA) according to the manufacturer’s instructions. We used DNaseI for DNA digestion. The NanoDrop^®^ ND-2000 (Thermo Scientific, USA) and Agilent 2100 Bioanalyzer with Agilent RNA 6000 Nano Kit were used for RNA quality assessment. Messenger RNA (mRNA) was purified from total RNA using poly-T oligo-attached magnetic beads followed by RNA purification and fragmentation (over 90% removal efficiency). The cDNA libraries for sequencing were constructed using Illumina TruSeq Stranded Kit (Illumina, USA) following the manufacturer’s protocol. An Illumina Hiseq 2500 platform was used to sequence the cDNA libraries [53, 54].

### Transcriptomic data analysis

Reads with low quality, adaptors, and a high proportion of unknown bases (> 5%) were removed to obtain clean reads. We used the short oligonucleotide alignment program (SOAP) and the hierarchical indexing for spliced alignment of transcripts (HISAT) to align and compare clean reads with the *E. harbinense* genome [3, 55, 56] and the Coding Potential Calculator (CPC) to predict the protein-coding ability of newly defined transcripts [54, 57]. For gene expression analysis, Bowtie2 was used to align the reads with the *E. harbinense* genome, and the RNASeq by Expectation Maximization (RSEM) package was used to calculate gene expression levels [58]. The coverage, reads distribution, and sequencing saturation of genes were evaluated based on gene expression data. The ade4 package in R software was used for principal component analysis (PCA). Hclust function in R software with the Euclidean distance algorithm was used for cluster tree analysis. Weighted Gene Co-Expression Network Analysis (WGCNA) was used for analysis of the gene co-expression network [59].

DESeq2 was used to detect differentially expressed genes (DEGs) (log_2_(FoldChange) > 1, *p* value < 0.05) [60]. Gene ontology analysis was performed by assigning Gene Ontology (GO) database categories with the Kyoto Encyclopedia of Genes and Genomes (KEGG) database [61]. The python package Pycluster was employed for hierarchical clustering analysis of DEGs using R package clusterProfiler for KEGG enrichment. We used PSORTb 3.0.2 for prediction of protein subcellular localization [11].

## Supplementary information

**Additional file 1: Table S1.** DEGs involved in the special pathways of *E. harbinense* under different pH conditions. **Table S2.** KEGG pathway analysis of differentially expressed new transcripts in *E. harbinense* strains involved in pH response. **Figure S1.** The pH changes in *E. harbinense* fermentation broth under different initial pH conditions. **Figure S2.** Correlation analysis and expression distribution of *E. harbinense* transcriptomes under different initial pH conditions. **Figure S3.** Gene co-expression network analysis of *E. harbinense* transcriptomes under different initial pH conditions. **Figure S4.** Differentially expressed genes of *E. harbinense* involved in the PTS system. **Figure S5.** Differentially expressed new transcripts of *E. harbinense* involved in pH response.

## Data Availability

The datasets supporting the conclusions of this article are included within the article and its additional file.

## Abbreviations

ETF: Ethanol-type fermentation
DEGs: Differentially expressed genes
PTS: Phosphotransferase system
AD: Anaerobic digestion
ORP: Oxidation reduction potential
CSTR: Continuous stirred-tank reactor
PYG medium: Peptone/yeast extract/glucose medium
SOAP: Short oligonucleotide alignment program
HISAT: Hierarchical indexing for spliced alignment of transcripts
CPC: Coding potential calculator
RSEM: RNASeq by expectation maximization
PCA: Principal component analysis
WGCNA: Weighted gene co-expression network analysis
GO: Gene ontology
KEGG: Kyoto Encyclopedia of Genes and Genomes
PFOR: Pyruvate:ferredoxin (flavodoxin) oxidoreductase
PtsA: Phosphoenolpyruvate–protein phosphotransferase
