# Supplementary material for: Metabolic regulation of ethanol-type fermentation of anaerobic acidogenesis at different pH based on transcriptome analysis of Ethanoligenens harbinense
Source: Biotechnol Biofuels. 2020 Jun 3;13:101. doi: 10.1186/s13068-020-01740-w (PMC7268672; doi:10.1186/s13068-020-01740-w)
Supplement: Supplementary file 1 — Additional file 1: Table S1. DEGs involved in the special pathways of E. harbinense under different pH conditions. Table S2. KEGG pathway analysis of differentially expressed new transcripts in E. harbinense strains involved in pH response. Figure S1. The pH changes in E. harbinense fermentation broth under different initial pH conditions. Figure S2. Correlation analysis and expression distribution of E. harbinense transcriptomes under different initial pH conditions. Figure S3. Gene co-expression network analysis of E. harbinense transcriptomes under different initial pH conditions. Figure S4. Differentially expressed genes of E. harbinense involved in the PTS system. Figure S5. Differentially expressed new transcripts of E. harbinense involved in pH response. [file 13068_2020_1740_MOESM1_ESM.docx]

**Additional information**

**Adaptation of ethanol-type fermentation of anaerobic acidogenesis to low pH based on transcriptome analysis of *Ethanoligenens***

Zhen Li, Yu Lou, Jie Ding, Bingfeng Liu, Guo-jun Xie, Nanqi Ren, Defeng Xing*

State Key Laboratory of Urban Water Resource and Environment, School of Environment, Harbin Institute of Technology, Harbin 150090, China

***Corresponding author.** School of Environment, P.O. Box 2614, 73 Huanghe Road, Nangang District, Harbin, Heilongjiang Province 150090, China

E-mail address: [dxing@hit.edu.cn](mailto:dxing@hit.edu.cn); Tel: +0086-451-86283123

**Table S1** DEGs involved in the special pathways of *E. harbinense* under different pH conditions.

| Gene ID | Ontoloty | GO term | Gene function | pH4/pH7 | pH5/pH7 | pH6/pH7 | pH8/pH7 |
| --- | --- | --- | --- | --- | --- | --- | --- |
| Ethha_1123 | biological process  ＆ molecular function | response to stimulus and signaling | HAMP domain-containing protein | -1.03 | 0.00 | 0.00 | 0.00 |
| Ethha_1927 |  |  | HAMP domain-containing histidine kinase | 1.57 | 1.46 | 0.00 | 0.00 |
| Ethha_2543 | biological process  ＆ molecular function | response to stimulus, locomotion and signaling | chemotaxis response regulator protein-glutamate methylesterase CheB | -1.85 | -2.47 | 0.00 | -2.08 |
| Ethha_2545 |  |  | purine-binding chemotaxis protein CheW | -2.01 | -2.44 | 0.00 | -2.23 |
| Ethha_2546 |  |  | chemotaxis protein CheA | -1.74 | -1.86 | 0.00 | -2.20 |
| Ethha_2541 | biological process | biological adhesion | flagellar filament capping protein FliD | -1.93 | 0.00 | 0.00 | -3.13 |
| Ethha_2578 | biological process | response to stimulus and locomotion | flagellar motor switch protein FliG | -2.44 | -1.83 | 0.00 | -1.35 |
| Ethha_2553 | biological process | locomotion | flagellar hook-associated protein FlgK | -2.06 | 0.00 | 0.00 | -2.54 |
| Ethha_2556 |  |  | flagellar hook-basal body protein | -1.13 | 0.00 | 0.00 | 0.00 |
| Ethha_2557 |  |  | flagellar hook basal-body protein | -1.32 | 0.00 | 0.00 | 0.00 |
| Ethha_2567 |  |  | flagellar motor switch protein FliM | -1.64 | -2.78 | 0.00 | -1.44 |
| Ethha_2570 |  |  | flagellar hook-basal body complex protein | -2.33 | -2.94 | 0.00 | -1.38 |
| Ethha_2575 |  |  | flagellar protein export ATPase FliI | -2.69 | -2.93 | 0.00 | -1.38 |
| Ethha_2579 |  |  | flagellar M-ring protein FliF | -2.44 | -1.62 | 0.00 | -1.09 |
| Ethha_2581 |  |  | flagellar basal body rod protein FlgC | -2.47 | -1.00 | 0.00 | 0.00 |
| Ethha_2580 |  |  | flagellar hook-basal body complex protein FliE | -2.55 | -1.31 | 0.00 | 0.00 |
| Ethha_2548 |  |  | flagellin | -2.31 | -1.46 | 0.00 | -4.56 |
| Ethha_2772 | biological process  ＆ molecular function | response to stimulus, detoxification and antioxidant activity | glutathione peroxidase | -3.57 | 0.00 | 0.00 | -2.15 |
| Ethha_2703 | biological process | response to stimulus, detoxification | desulfoferrodoxin | -1.41 | 0.00 | 0.00 | -1.00 |
| Ethha_0300 | molecular function  ＆ biological process | response to stimulus, antioxidant activity and detoxification | peroxiredoxin | 2.12 | 3.45 | 2.86 | 2.35 |
| Ethha_0354 |  |  | thioredoxin-disulfide reductase | 1.04 | 0 | 0 | 0 |
| Ethha_1213 |  |  | divergent PAP2 family protein | -1.23 | -1.65 | 0 | 0 |
| Ethha_1510 |  |  | carboxymuconolactone decarboxylase family protein | -1.07 | 0 | 0 | 1.22 |
| Ethha_1399 |  |  | redoxin domain-containing protein | 2.14 | 2.09 | 0 | 1.00 |
| Ethha_0425 | molecular function | electron carrier activity | FMN-dependent NADH-azoreductase | 3.67 | 3.11 | 0.00 | 1.89 |
| Ethha_1060 |  |  | rubredoxin | -1.07 | 0.00 | 0.00 | -1.24 |
| Ethha_1384 |  |  | thioredoxin family protein | -1.95 | 1.45 | 0.00 | 0.00 |
| Ethha_1504 |  |  | nitric oxide synthase | 2.93 | 1.83 | 1.31 | 1.56 |
| Ethha_1933 |  |  | ferredoxin | -2.83 | -2.91 | 0.00 | -3.74 |

**Table S2** KEGG pathway analysis of differentially expressed new transcripts in *E. harbinense* strains involved in pH response.

| Gene | Number of KEGG ID | Pathway level 1 | Pathway level 2 |
| --- | --- | --- | --- |
| BGI_novel_C02 | 2 | Metabolism | Metabolism of terpenoids and polyketides |
| BGI_novel_C12 | 1 | Environmental Information Processing | Membrane transport |
| BGI_novel_C16 | 1 | Genetic Information Processing | Replication and repair |
| BGI_novel_C19 | 1 | Environmental Information Processing | Membrane transport |
| BGI_novel_C20 | 2 | Cellular Processes | Cellular community-prokaryotes and Cell growth and death |
| BGI_novel_C28 | 4 | Cellular Processes | Cell motility |
|  |  | Environmental Information Processing | Signal transduction |
|  |  | Metabolism | Global and overview maps and Carbohydrate metabolism |
| BGI_novel_C31 | 11 | Metabolism | Global and overview maps; Carbohydrate metabolism; Nucleotide metabolism; Biosynthesis of other secondary metabolites |



 **Figure S1.** The H_2_ contents (a) and the pH changes in fermentation broth (b) of *E. harbinense* under different initial pH conditions.


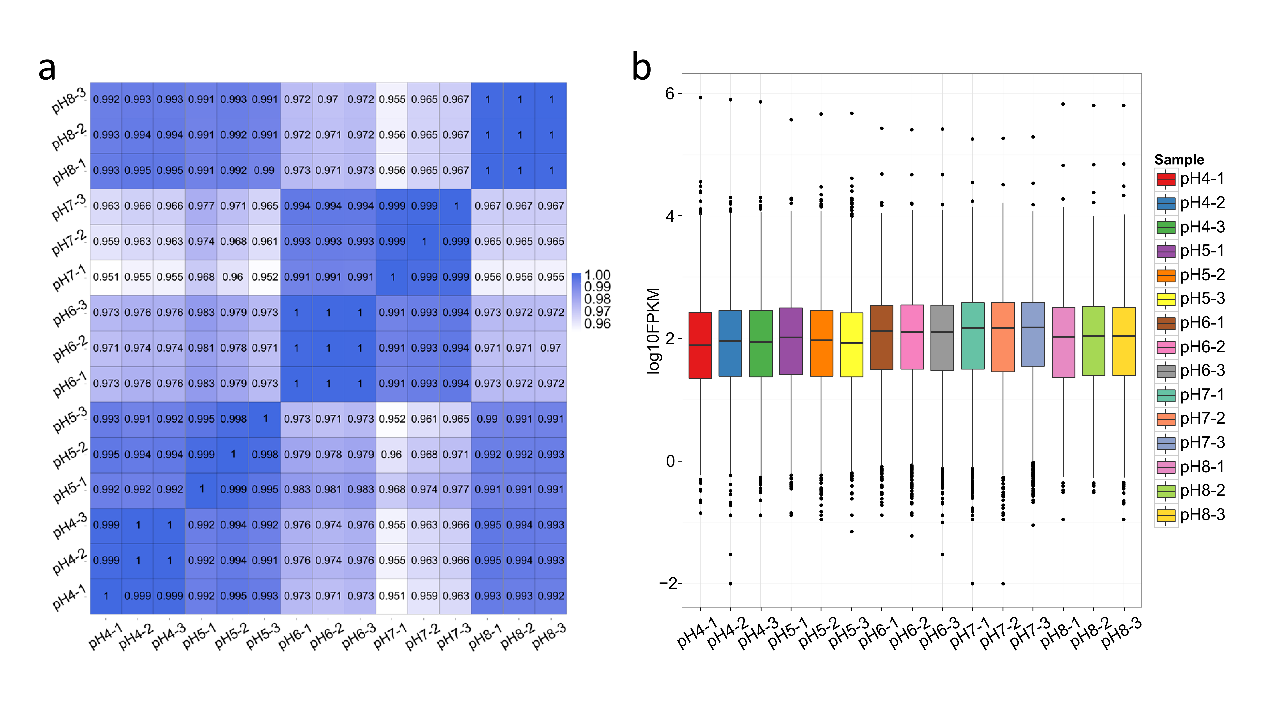


**Figure S2.** Correlation analysis and expression distribution of *E. harbinense* transcriptomes under different initial pH conditions.

**
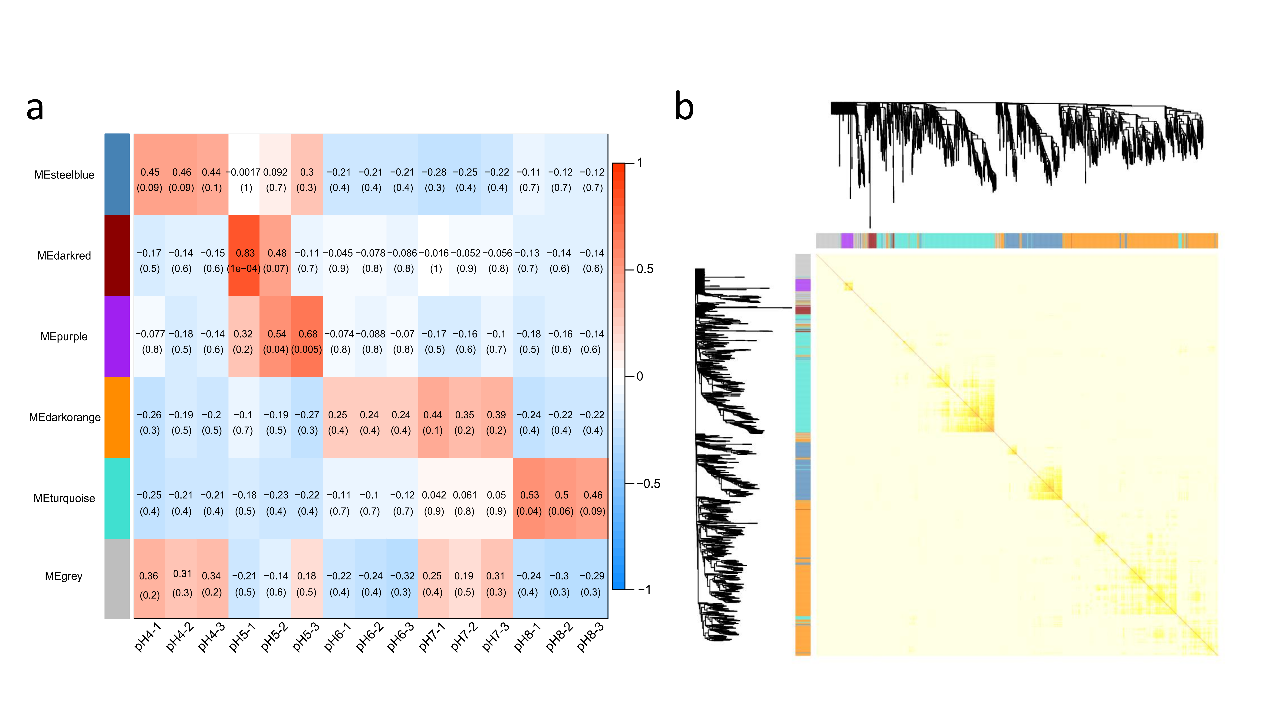
**

**Figure S3.** Gene co-expression network analysis of *E. harbinense* transcriptomes under different initial pH conditions.

**

**

**Figure S4.** Differentially expressed genes of *E. harbinense* involved in the PTS system.





**Figure S5.** Differentially expressed new transcripts of *E. harbinense* involved in pH response.
